# Surgical treatment of giant phyllodes tumors of the breast: a series of rare cases

**DOI:** 10.3389/fonc.2025.1591306

**Published:** 2025-10-16

**Authors:** Chang Chen, Ying Xu, Xin Huang, Yan Lin, Qiang Sun

**Affiliations:** Department of Breast Surgery, Peking Union Medical College Hospital, Chinese Academy of Medical Sciences and Peking Union Medical College, Beijing, China

**Keywords:** breast, phyllodes tumor, surgery, giant tumor, rare cases

## Abstract

**Background:**

Phyllodes tumors (PTs) are rare interstitial tumors that account for <1% of all breast tumors. The best surgical option for PTs is controversial, particularly in patients with giant PTs.

**Methods:**

This retrospective single-center study selected patients with giant borderline/malignant PT (>10 cm) treated in our center between January 2017 and July 2022. We conducted a detailed analysis and identified rare cases. Survival analysis was performed using the Kaplan–Meier method.

**Results:**

This study included 24 patients with PTs >10 cm, including 15 borderline and nine malignant PTs. Among these patients, 12 underwent extended lumpectomy and 12 underwent mastectomy at initial diagnosis. All patients were followed up for a median of 49.8 months. Local recurrence occurred in 50% of the patients who underwent lumpectomy, whereas none of the patients who underwent mastectomy and achieved R0 resection had recurrence. Several special cases are described in detail separately.

**Conclusion:**

Mastectomy may reduce local recurrence rates in patients with giant borderline/malignant PTs of the breast. Complete tumor resection and clean margins are the most important factors in controlling local recurrence.

## Introduction

Phyllodes tumors (PTs) of the breast are rare interstitial tumors derived from the fibroepithelial cells of the breast and account for <1% of breast tumors (1). Pathologically, PTs are mostly composed of fibrous connective and epithelial tissue of the breast and are categorized into benign, borderline, and malignant subtypes according to the tumor cell characteristics, such as cell atypia, mitosis proportion, and tumor necrosis (2, 3). Borderline/malignant PTs have a high recurrence rate (4, 5). Among malignant tumors, the metastasis rate can reach 10.0%–40.0% (6).

Clinically, the peak patient age for PTs is approximately 40 years. The tumor usually presents as a single painless lobulated mass. In most cases, the tumor grows continuously throughout the disease course. However, it may present as a rapidly increasing neoplasm and may even occupy the entire breast. Extended lumpectomy with negative margins is now widely accepted as a proper surgical procedure, and some scholars also recommend mastectomy if a negative margin cannot be guaranteed (7–9). Currently, whether the local recurrence rate is higher in patients who undergo lumpectomy than in those who undergo mastectomy remains controversial (10–12), especially in patients with large borderline/malignant tumors. The appropriate margin width also remains debated (6). Owing to the rarity of the disease, especially the low number of malignant and borderline subtypes, randomized controlled trials have not been conducted. Thus, the optimal treatment is uncertain, particularly for patients with large tumors. A phyllodes tumor measuring more than 10 cm in diameter is usually defined as a “giant” tumor (13, 14), therefore, we analyzed the surgical methods and postoperative follow-up results in patients with borderline and malignant PTs >10 cm in our center over the past six years to explore the optimal treatment options and prognosis for this group of patients.

## Methods

This retrospective, single-center case series presents the clinical and histopathological descriptions of a series of patients with giant borderline/malignant PT (>10 cm) treated at our center between January 2017 and July 2022. The inclusion criteria included: definite pathological diagnosis of borderline or malignant PT, a diameter exceeding 10 cm, and completion of diagnosis, treatment, and follow-up at our institution. Exclusion criteria included: tumors with a diameter less than 10 cm, benign PT, and patients with incomplete clinical data. All results were independently diagnosed by two pathologists. The surgical plan was decided by the surgeon together with the patient based on the patient’s wishes, along with a combination of factors such as the mass and breast size. Unless otherwise specified, none of the patients who underwent extended lumpectomy or mastectomy had residual tumors.

The authors received approval from the Ethics Committee of Peking Union Medical College Hospital. This study was conducted in accordance with the Declaration of Helsinki of 1964 and its subsequent amendments. All participants provided informed consent for inclusion in the study and its publication. This case series was reported in accordance with the PROCESS guidelines (15).

The primary outcome was disease-free survival (DFS), which was measured from the date of PT diagnosis to either the recorded date of recurrence, metastasis, or the date of last contact. DFS was estimated using the Kaplan–Meier method and compared using the log-rank test. Statistical analysis was conducted using IBM SPSS Statistics for Windows, version 22.0, with p<0.05 indicating statistical significance.

## Results

### Patient characteristics

A total of 24 patients with PTs >10 cm was finally included, including 15 borderline and nine malignant tumors. Among these, 12 patients underwent extended lumpectomy, and 12 underwent mastectomy at initial diagnosis. Information on the patients who chose different surgical methods is shown in **Table 1**, while the detailed information on each patient is shown in **Table 2**. Only one patient in the mastectomy group had a preserved nipple areola. None of the patients underwent flap or skin grafting. Among patients who underwent extended lumpectomy, most had tumors <20 cm (91.7%). Most patients with tumors measuring >20 cm underwent total mastectomy (85.7%, 6/7). At the time of this report, 50% of patients who underwent extended lumpectomy had local recurrence, while all but one patient (who developed local progression due to incomplete resection) who underwent mastectomy did not develop local recurrence. Here, we describe several patients in detail with photographs.

**Table 1:** Characteristics of patients with different surgical methods.

Modus operation	Breast-conserving surgery	Mastectomy	Total
	N=12 (50%)	N=12 (50%)	N=24 (100%)
Age (years)			
Mean	41.6	42	41.8
Range	18-52	32-69	18-69
Pathological subtypes			
Borderline	7 (58.3)	8(66.7)	15 (62.5)
Malignant	5 (41.7)	4 (33.3)	9 (37.5)
Tumor size (cm)			
10≤T<20	11 (91.7)	6 (50.0)	17 (70.8)
T≥20	1 (8.3)	6 (50.0)	7 (29.2)
Recrudescence	6 (50.0)	1 (8.3)	7 (29.2)
DFS (months, mean)	10.3	–	10.3

DFS, disease free survival.

**Table 2:** Detailed information of all patients.

No.	Age*	Pathology grade	Tumor size (cm)	Type of first breast surgery	Axillary surgery	Lymph node status	Post-operative adjuvant therapy	Recurrence	DFS (months)
1	45	Malignant	26	Mastectomy	None	NA	None	Yes	1
2	52	Malignant	13	Extended lumpectomy	None	NA	None	No	157
3	46	Malignant	12	Extended lumpectomy	None	NA	None	Yes	12
4	38	Malignant	14	Extended lumpectomy	None	NA	None	No	140
5	45	Borderline	15	Extended lumpectomy	None	NA	None	No	137
6	38	Borderline	13	Mastectomy	None	NA	None	No	137
7	38	Borderline	13	Extended lumpectomy	None	NA	None	Yes	24
8	45	Borderline	18	Mastectomy	SLNB	Negative	None	No	126
9	36	Borderline	25.5	Mastectomy	ALND	Negative	None	No	125
10	33	Borderline	13	Extended lumpectomy	None	NA	None	No	121
11	36	Malignant	20	Extended lumpectomy	None	NA	None	Yes	6
12	69	Malignant	10	Mastectomy	SLNB	Negative	None	No	107
13	33	Borderline	10	Extended lumpectomy	None	NA	None	No	104
14	25	Borderline	14	Extended lumpectomy	None	NA	None	No	81
15	41	Borderline	15.2	Mastectomy	SLNB	Negative	None	No	52
16	52	Borderline	25	Mastectomy	SLNB	Negative	None	No	48
17	18	Malignant	11	Extended lumpectomy	None	NA	None	Yes	2
18	32	Malignant	21.5	Mastectomy	ALND	Positive	Chemotherapy	No	4
19	47	Borderline	14	Extended lumpectomy	None	NA	None	Yes	6
20	52	Borderline	25	Mastectomy	ALND	Negative	None	No	20
21	51	Malignant	48	Mastectomy	ALND	Negative	None	No	19
22	52	Borderline	16	Mastectomy	SLNB	Negative	None	No	4
23	37	Borderline	13	Extended lumpectomy	None	NA	None	Yes	12
24	34	Borderline	15.5	Nipple-sparing mastectomy	None	NA	None	No	60

DFS, disease free survival; SLNB, sentinel lymph node excision biopsy; ALND, axillary lymph node dissection; NA, not available.

*Age at first diagnosis

### Case presentations

#### Case 1

**Figure 1:**
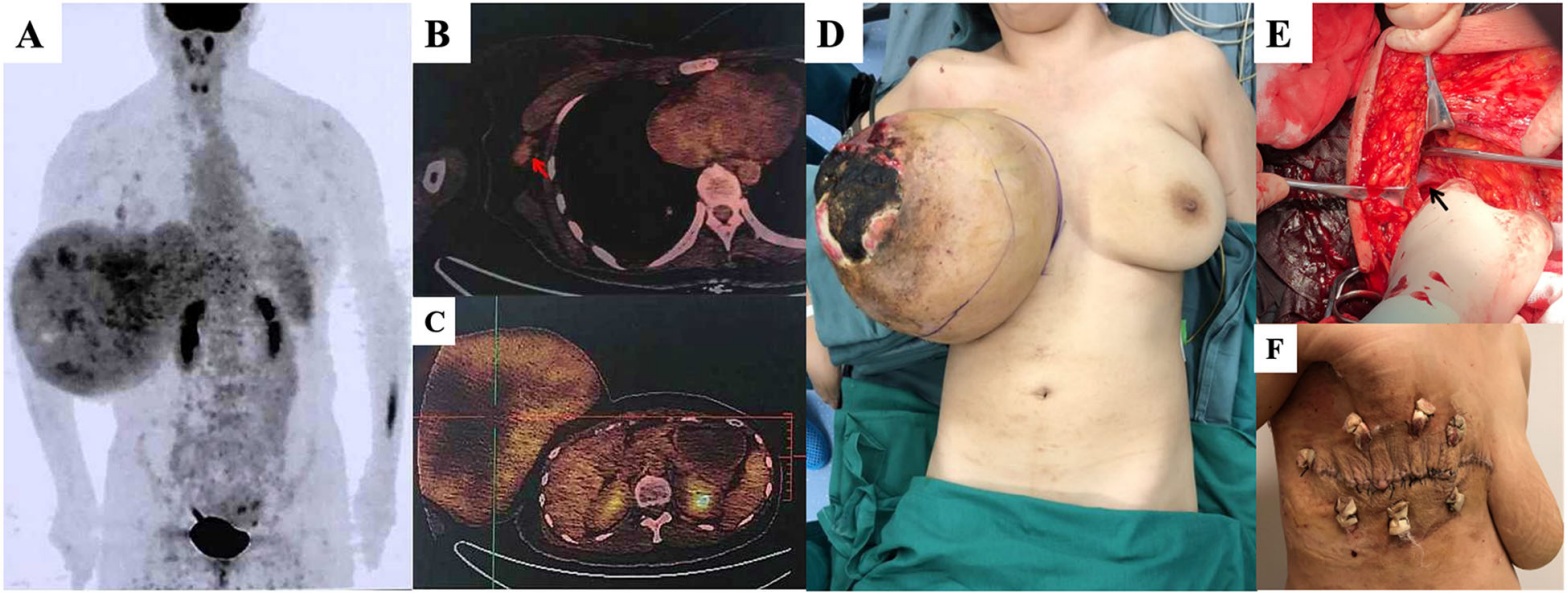
**(A)** A tumor with a maximum sectional area of 24 x 16cm by positron emission tomography/computed tomography (PET/CT) scan, with no abnormality in the lungs or bone. **(B)** Enlarged lymph nodes (red arrow) in the right axilla, measuring 0.4-1.2 cm and with a maximum standardized uptake value (SUVmax) of 2.1. **(C)** Uneven increase the SUV of the mass, with an SUVmax value of 3.4. **(D)** The patient was placed under general anesthesia in the supine position. A giant right breast tumor with skin ulceration is visible. **(E)** The axillary lymph nodes were dissected (black arrow: axillary vein). **(F)** Surface wound 14 days after surgery.

A 36-year-old woman presented with a rapidly enlarging right breast mass. The tumor had been present for several years, but its growth accelerated over the past two months, culminating in tumor rupture after taking Chinese medicine in a local hospital. Physical examination revealed an ellipsoidal mass occupying the entire right breast, which was hard with poor mobility, without tenderness, and a diameter of approximately 30 cm (**Figure 1**). Skin ulceration with an area of approximately 15 × 15 cm was observed in the external quadrant of the right breast, with pus moss and a putrefactive odor. Palpation revealed several enlarged lymph nodes in the right axilla with good mobility. Positron emission tomography/computed tomography (PET/CT) suggested a maximum sectional area of the mass of approximately 24 × 16 cm, with unevenly increasing standardized uptake value (SUV) and a maximum value (SUVmax) of 3.4. Enlarged lymph nodes were observed in the right axilla, as well as the deep side of the pectoralis major, ranging in size from 0.4–1.2 cm and an SUVmax of 2.1. No abnormalities were detected in other areas such as the lungs and bones. The patient underwent a modified radical mastectomy. The tumor boundaries were clear, and no infiltration of the surrounding tissue was observed by naked eye. Gross pathological examination indicated that the right breast weighed 7.5 kg and measured 28 × 28 × 17.5 cm. The mass measured 25.5 × 20 × 22 cm after incision. Microscopic pathology suggested that the mass was consistent with borderline breast PT and was proximate to the bottom of the cutting edge. The lymph nodes showed chronic inflammation (axillary lymph node: 0/29, third station lymph node: 0/6). The immunohistochemical results showed CD117 (-), P16 (+), Ki-67 (index 10%), P53(-), and phosphohistone H3 (PHH3, positive cells: 1/10 hpf). The patient was followed up four years after surgery, and no local recurrence or distant metastasis was found.

#### Case 2

**Figure 2:**
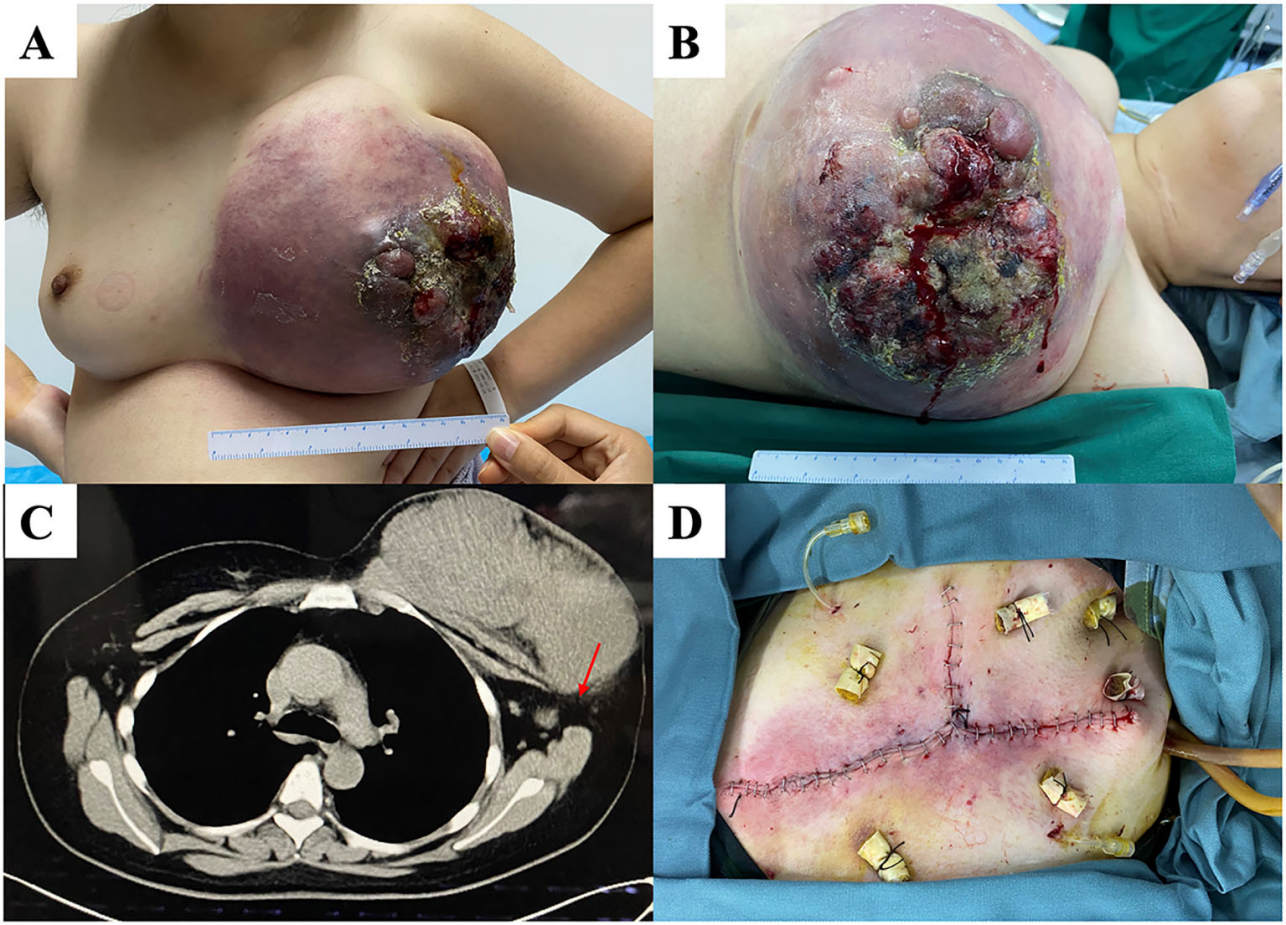
**(A, B)** A patient with a huge tumor occupying the whole left breast with a broken surface. **(C)** Chest computed tomography (CT) showing that the is closely tumor adhered to partially invading the chest wall. Enlarged lymph nodes (red arrow) are present. **(D)** We performed modified radical mastectomy, preserving as much of the normal skin tissue as possible.

A 32-year-old woman had a left breast tumor that had persisted for more than five years. The tumor diameter was initially 2 cm but had increased to >20 cm at the time of the hospital visit (**Figure 2**). The patient underwent a core needle biopsy of the tumor before visiting our hospital, and the pathology showed a malignant PT. After admission, chest computed tomography (CT) revealed a left breast mass adhered to the chest wall and abnormal enlargement of the axillary lymph nodes. PET/CT indicated high metabolism in the axillary lymph nodes but no distant metastasis. Therefore, we performed a modified radical mastectomy. During the operation, we removed part of the pectoral muscle because of tumor invasion. Pathological examination confirmed that the lesion was a malignant PT with lymph node metastasis involving the skin tissue, with tumor observed at part of the cutting edge. The maximum tumor was 21.5 cm. The immunohistochemical results showed AE1/AE3 (partial+), CD31 (vascular+), CD34 (vascular+), Ki-67 (index 30%), Myo-D1 (-), smooth muscle actin (SMA) (-), and S-100 (-). Considering that the tumor could be seen at the cutting edge of the patient and the lymph node had metastasis, we suggested that the patient undergo radiotherapy and chemotherapy (anthracycline and cyclophosphamide) after surgery. However, the patient did not receive further treatment in our hospital and was lost to follow-up.

#### Case 3

**Figure 3:**
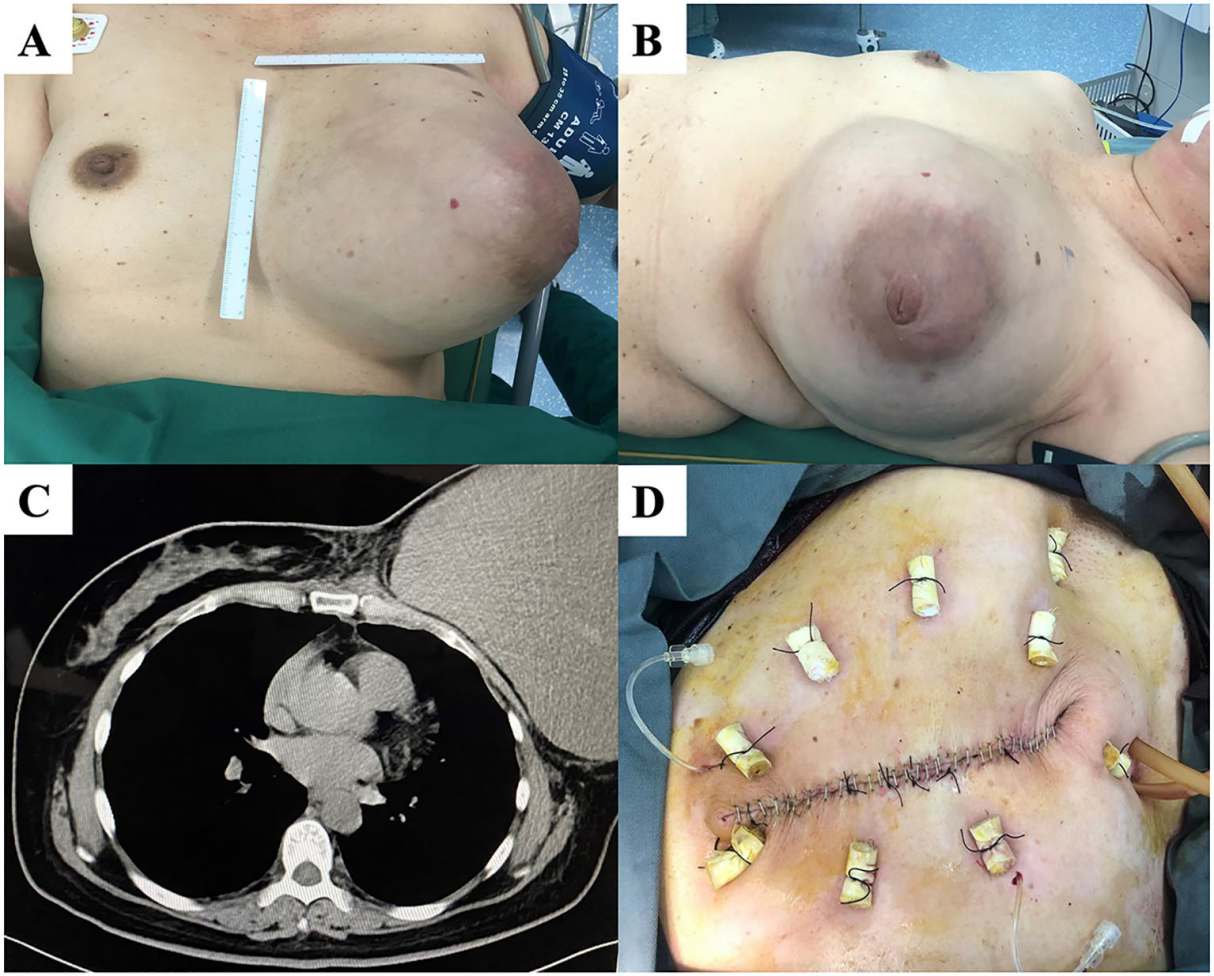
**(A, B)** A patient with a large left breast mass and intact and unbroken skin. **(C)** Chest computed tomography (CT) showing a clear demarcation of the mass from the chest wall. **(D)** Skin sutured after total mastectomy.

A left breast lump was incidentally discovered five years before a 52-year-old woman presented to our department. The patient did not receive any specific treatment until her left breast lump gradually increased in size to >20 cm. Her left breast mass occupied the entire breast when she visited the outpatient department (**Figure 3**). Chest CT showed a clear boundary and complete capsule between the left breast mass and the chest wall. Subsequently, mastectomy was performed. Postoperative pathology showed that the tumor was a borderline PT with a negative margin and a maximum diameter of 25 cm. The immunohistochemical results were Ki-67 (hot spot index 20%), SMA (+), CD34 (+), S-100 (-), and AE1/AE3 (-). The patient recovered well after surgery and was followed up for one and a half years, with no local recurrence.

#### Case 4

**Figure 4:**
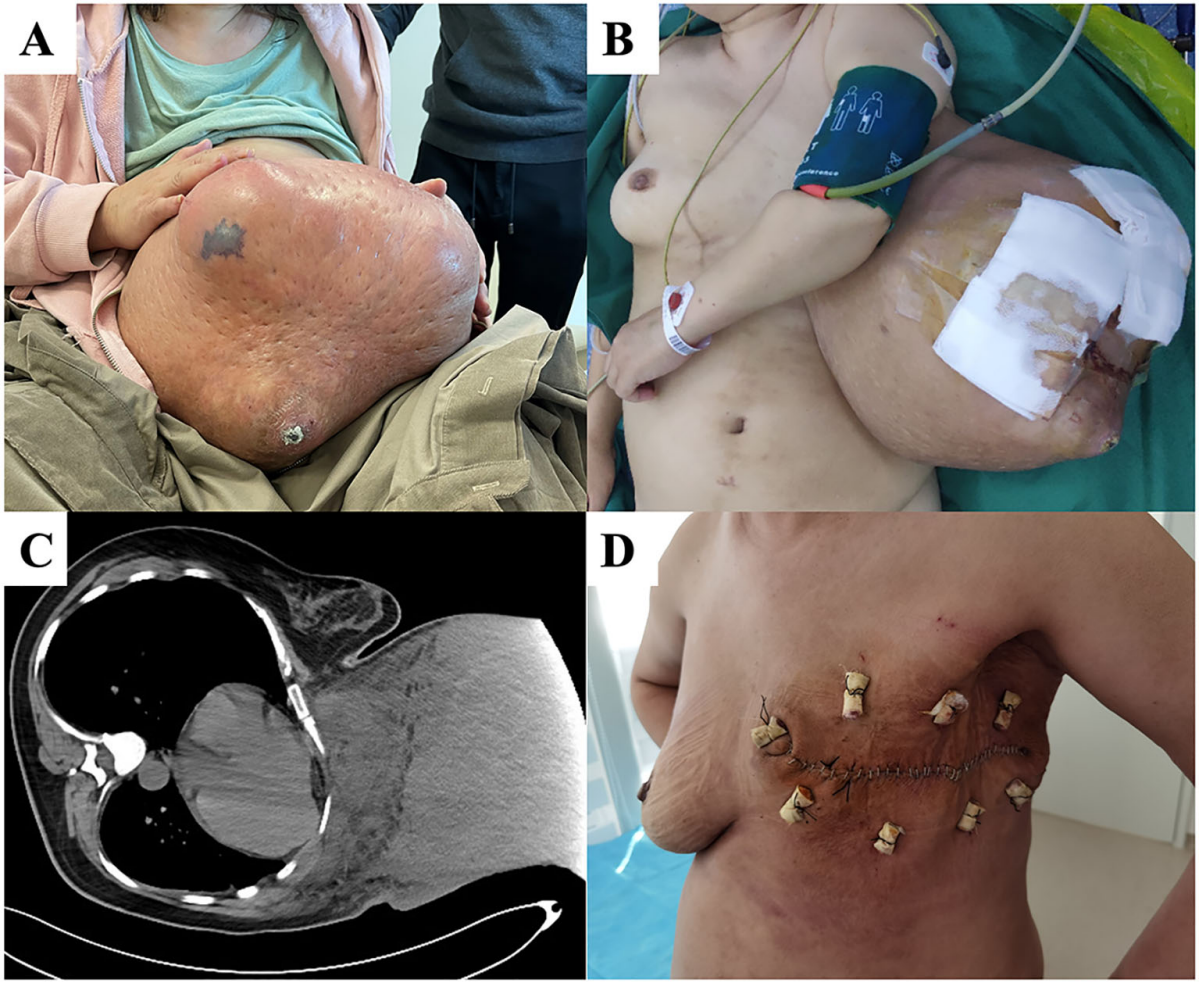
**(A)** At the time of the first visit, the patient was unable to walk and could not sleep flat on her back due to a huge left breast mass. **(B)** After general anesthesia, the patient was placed in a flat position and the local skin breakdown was covered with gauze. **(C)** Chest computed tomography (CT) showing tight adhesion of the mass to the chest wall with localized invasion. **(D)** The patient’s wound healed well 14 days after surgery.

A 51-year-old woman found a left breast mass two years before her presentation to our department. The tumor gradually increased in size over two years and a period of accelerated tumor growth was observed temporally associated with the patient’s intake of Chinese herbal medicine. One month before she visited our hospital, she was unable to sleep flat on her back and had difficulty walking (**Figure 4A**). Examination showed a tumor >30 cm occupying her left breast. The skin on the surface was partially broken, and palpation showed enlarged and hard lymph nodes in the left axilla. After admission, PET/CT indicated increased metabolism in the left breast mass, left axillary lymph node, and left subclavian lymph node, suggesting malignancy. Therefore, we performed a modified radical surgery for the left breast tumor. Postoperative pathology revealed a malignant PT with a maximum diameter of 48 cm, invading the surrounding skin but with negative cut margins. The lymph nodes showed signs of chronic inflammation. The immunohistochemical results were SMA (focal +), Desmin (-), CD34 (partial +), S-100 (-), Ki-67 dense (index 40%), AE1/AE3 (-), epithelial membrane antigen (EMA) (-), SRY-related HMG-box 10 protein (SOX10) (-), signal transducer and activator of transcription 6 (STAT6) (-), and special AT-rich sequence-binding protein 2 (SATB2) (-). The patient’s wound healed well after surgery (**Figure 4D**). No recurrence or metastasis was observed at the one-year follow-up and to date.

#### Case 5

**Figure 5:**
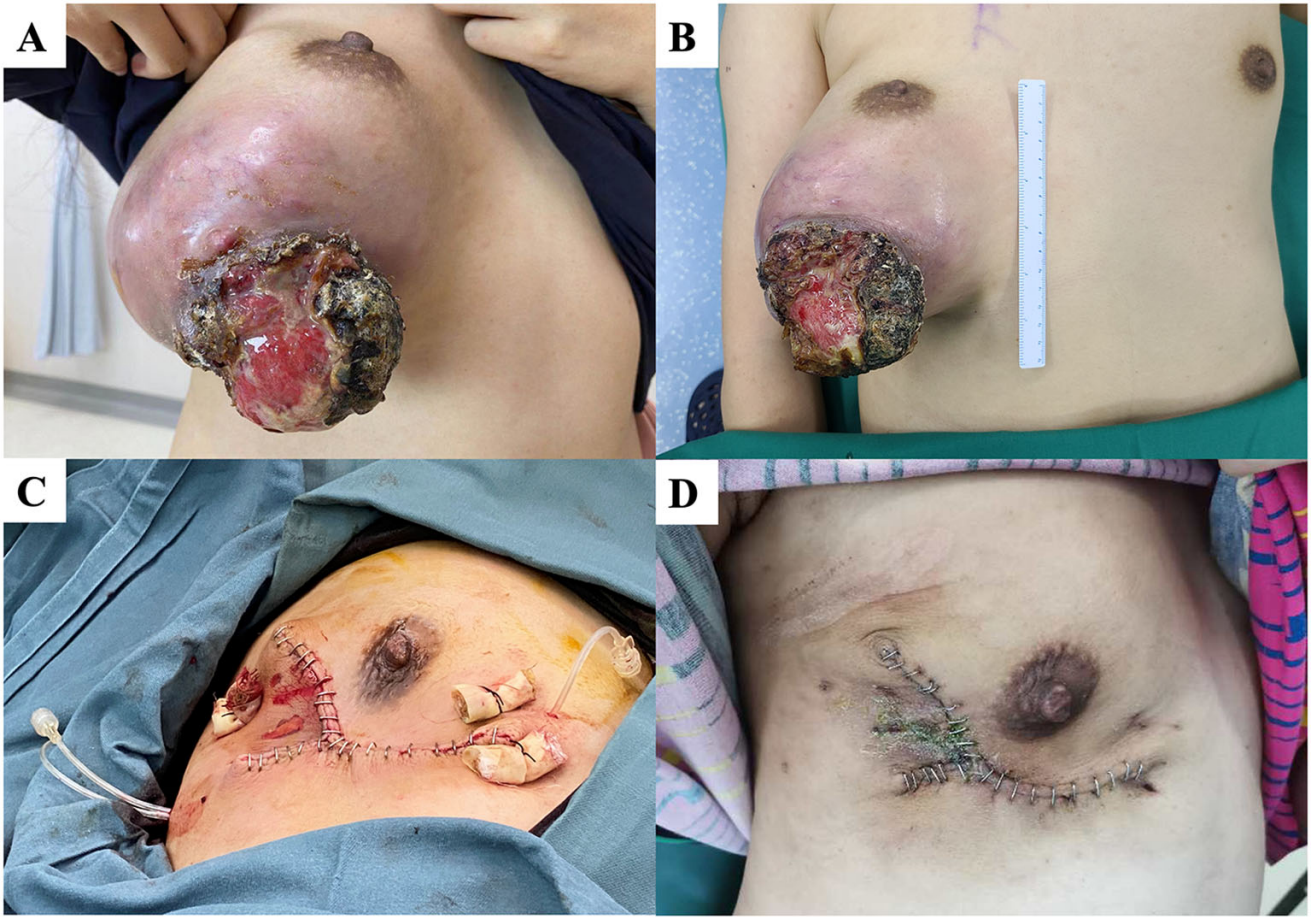
**(A, B)** A patient with a large mass under the right external breast with localized rupture and ulcers. **(C)** We performed nipple-sparing mastectomy. **(D)** The patient’s wound healed well 14 days after surgery.

A 34-year-old woman had a right breast mass for more than five years. The tumor increased rapidly in size before arriving at our hospital, and the surface broke down into a cauliflower-shaped shape. On admission, a large mass was observed in the lower part of the right outer breast with visible ulceration but no involvement of the nipple areola (**Figures 5A, B**). Preoperative chest CT showed that the tumor had not invaded the chest wall. We performed nipple-sparing mastectomy (**Figure 5C**), and the patient recovered well postoperatively (**Figure 5D**). Pathology showed that the tumor measured 15.5 × 11 × 10.5 cm and was a borderline PT. Two and a half years postoperatively, no local recurrence has been observed at follow-up.

### Follow-up and prognosis

**Figure 6:**
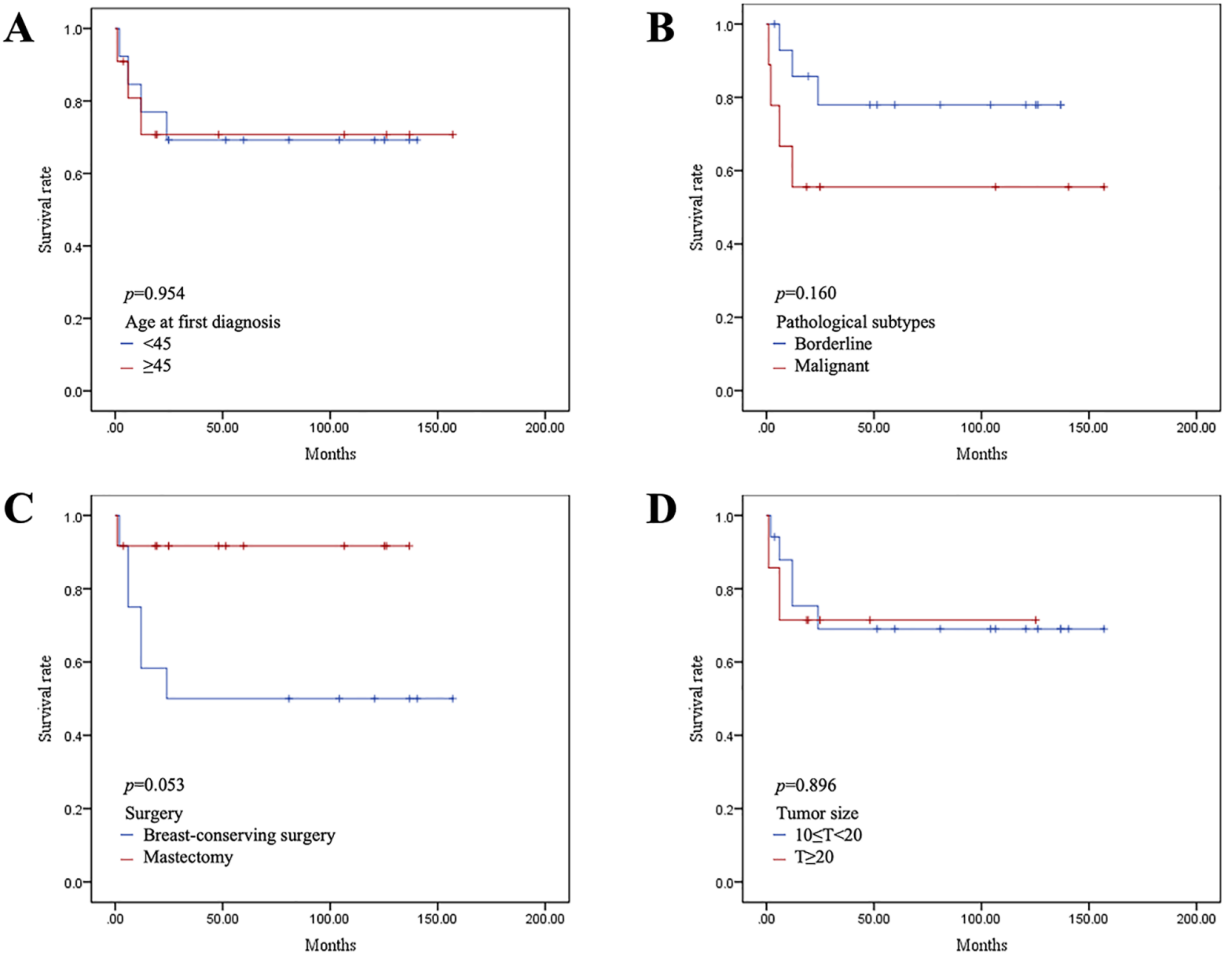
Kaplan–Meier survival curves of disease-free survival (DFS) according to age at diagnosis **(A)**, pathological type **(B)**, surgical method **(C)**, and tumor size **(D)**.

All patients were followed up for a median of 49.8 months. Local recurrence occurred within two years after the operation. We performed mastectomy for recurrent tumors and lumpectomy for the first time. None of the patients experienced relapse. Of the patients who underwent mastectomy, only Case 2 experienced local progression due to a failure to achieve R0 resection. Although we recommended postoperative adjuvant radiotherapy and chemotherapy, she did not undergo further treatment at our hospital for personal reasons and was lost to follow-up several months later. The DFS analysis for all patients is shown in **Figure 6**. Patients with malignant PTs and those who underwent breast-conserving surgery had higher rates of local recurrence; however, no statistically significant difference was observed in DFS according to age, tumor type, surgical approach, or tumor size.

## Discussion

This study was a retrospective analysis of the surgical procedures and prognostic information of patients with giant PTs of the breast in recent years at our institution. But due to the limited sample size, this study is only an exploratory study. It was a rare collection of cases analyzed for giant tumor surgery.

The choice of surgical plan and determination of margin width for borderline and malignant PTs remains controversial, especially for patients with large tumors. Early studies concluded that mastectomy could reduce the risk of local recurrence compared with local excision of the mass. In 2008, Pezner et al. performed a retrospective study of 478 patients with malignant PTs and found that, after excluding the effect of postoperative adjuvant therapy, the five-year postoperative local control rate was 79.4% in patients who underwent local excision, compared with 91.2% in patients who underwent mastectomy (10). Although their study lacked information on the width of the margin in patients who underwent local excision, the multifactorial analysis of a retrospective study by Belkacémi et al. showed that mastectomy improved DFS in patients with borderline or malignant PTs after including factors such as margin status (16). However, Asoglu et al. found that extended local excision with adequate margins did not increase the local recurrence rate (7). In 2019, In their meta-analysis, Lu et al. showed that different surgical approaches did not influence the risk of local recurrence (11). However, their subgroup analysis revealed a significantly higher risk of local recurrence in patients with malignant PTs who underwent local extended excision compared to the risk in those who underwent mastectomy. The analysis based on the Surveillance, Epidemiology, and End Results (SEER) database suggested that patients with PTs who underwent local excision with wide negative margins did not have a statistically significant difference in overall survival compared to those who underwent mastectomy (4). Our data showed that for patients with tumors >10 cm in size, patients undergoing mastectomy showed a trend towards lower local recurrence.

The National Comprehensive Cancer Network (NCCN) guidelines recommend that the margin for local extended resection in patients with borderline or malignant PTs should be at least 1 cm; however, recent studies have reported no significant difference in postoperative local recurrence rate and survival between margin <1 cm and ≥1 cm, as long as the margin was negative. Rodrigues et al. divided patients with PTs into four groups according to their excision margin width (>1 cm, 0.2–1 cm, 0–0.2 cm, and positive margins) and reported a significantly higher local recurrence rate in the positive margin group. However, subgroup analysis suggested no statistically significant correlation between negative margin width and recurrence rate (17). Tremblay-LeMay et al. similarly concluded that a negative margin was critical, but it was only necessary to ensure that the negative margin width was >0.1 cm (6). In a cohort of 550 patients, a wider margin was not associated with a reduced risk of local recurrence (18). In that cohort, positive margins did not result in higher recurrence rates (18). Except for the risk of local recurrence, the margin status of borderline and malignant PTs had little effect on overall survival. Two recent meta-analyses showed no statistically significant differences in local recurrence rates and overall survival between margins <1 cm and > 1 cm in patients with borderline and malignant PTs (19, 20). Spitaleri et al. concluded that a positive surgical margin did not lead to a worse prognosis in patients with PTs (21). Information regarding the width of the incision margin was not included in the present study. However, the above-mentioned patients who underwent mastectomy all had margin widths <1 cm, but all showed good local control, except for those who did not achieve R0 resection. Uninvolved skin and tissue can be effectively preserved without intentionally pursuing a wider negative margin, and other invasive procedures such as skin grafting or flap transfer can be avoided.

Some PTs, especially borderline and malignant PTs, can rapidly increase in size, occupy the entire breast, and seriously affect patient quality of life. Surgical treatment remains the primary choice for these patients; however, surgery is difficult and requires a comprehensive multidisciplinary evaluation. In our experience, these patients must be fully evaluated preoperatively, and chest enhancement CT or magnetic resonance imaging (MRI) should be performed to fully assess tumor compression or invasion of the chest wall (22). Moreover, multidisciplinary surgery should be performed. Surgeons should pay attention to the complete resection of the entire tumor during surgery, avoiding incomplete resection or destroying the tumor integrity; otherwise, the tumor could recur in the short term (6, 17).

The incidence of axillary involvement is very low, occurring in only 1–2% of patients with malignant PT (23). The possibility of postoperative pathological metastasis is very low, even in patients with clinically enlarged axillary lymph nodes (23). Our data also confirmed that even when preoperative PET/CT or CT imaging suggested an abnormal enlargement of the axillary lymph nodes, few of the lymph nodes were metastatic after resection. Therefore, axillary lymph nodes should be carefully handled.

The largest limitation of this study was the small number of patients; therefore, high-quality survival analysis could not be performed and it was not possible to conduct a reliable multivariable analysis to adjust for confounding factor. Our data suggest that mastectomy may be associated with reduced recurrence risk in these giant tumors, but this requires confirmation in larger studies. The number of cases in our center is still increasing and will be analyzed further. Another limitation is that our pathological results did not include the width of the margin, which should be further improved in subsequent studies. Finally, owing to the small sample size of this retrospective and exploratory study, selection bias cannot be avoided. The fact that patients with larger tumors are more likely to undergo mastectomy may have an impact on outcomes (e.g., recurrence rate). This type of bias will need to be eliminated in future studies through statistical analysis following further expansion of the sample size.

## Conclusion

In patients with giant borderline/malignant PT of the breast, patients undergoing mastectomy showed a trend towards lower local recurrence; however, this difference did not reach statistical significance. Complete resection of the tumor and ensuring clean margins are the most important factors in controlling local recurrence. The findings of this study are specific to giant borderline and malignant phyllodes tumors exceeding 10 cm in diameter, and should not be extrapolated to smaller tumors.

## Data Availability

The original contributions presented in the study are included in the article/supplementary material. Further inquiries can be directed to the corresponding authors.
